# 30-day mortality after hip fracture surgery: Influence of postoperative factors

**DOI:** 10.1371/journal.pone.0246963

**Published:** 2021-02-16

**Authors:** Juan F. Blanco, Carmen da Casa, Carmen Pablos-Hernández, Alfonso González-Ramírez, José Miguel Julián-Enríquez, Agustín Díaz-Álvarez

**Affiliations:** 1 Trauma and Orthopaedics Department, University Hospital of Salamanca, Salamanca, Spain; 2 Instituto de Investigación Biomédica de Salamanca (IBSAL), Salamanca, Spain; 3 Orthogeriatric Unit, University Hospital of Salamanca, Salamanca, Spain; 4 Anaesthesiology Department, University Hospital of Salamanca, Salamanca, Spain; Assiut University Faculty of Medicine, EGYPT

## Abstract

**Purpose:**

The 30-day mortality rate after hip fracture surgery has been considered as an indirect indicator of the quality of care. The aim of this work is to analyse preoperative and postoperative factors potentially related to early 30-day mortality in patients over 65 undergoing hip fracture surgery.

**Methods:**

Prospective cohort study including all consecutive primary hip fracture patients over 65 admitted to Trauma and Orthopaedics department from January 1, 2018 to December 31, 2019. Bed-ridden, non- surgically treated patients, and high energy trauma or tumoral aetiology fractures were excluded. A total of 943 patients were eligible (attrition rate: 2.1%). Follow-up included 30-days after discharge. We noted the 30-day mortality after hip fracture surgery, analysing 130 potentially related variables including biodemographic, fracture-related, preoperative, and postoperative clinical factors. Qualitative variables were assessed by χ^2^, and quantitative variables by non-parametric tests. Odds ratio determined by binary logistic regression. We selected preventable candidate variables for multivariate risk assessment by logistic regression.

**Results:**

A total of 923 patients were enrolled (mean age 86.22±6.8, 72.9% women). The 30-day mortality rate was 6.0%. We noted significant increased mortality on men (OR = 2.381[1.371–4.136], p = 0.002), ageing patients (OR_year_ = 1.073[1.025–1.122], p = 0.002), and longer time to surgery (OR_day_ = 1.183[1.039–1146], p<0.001), on other 20 preoperative clinical variables, like lymphopenia (lymphocyte count <10^3^/μl, OR = 1.842[1.063–3.191], p = 0.029), hypoalbuminemia (≤3.5g/dl, OR = 2.474[1.316–4.643], p = 0.005), and oral anticoagulant intake (OR = 2.499[1.415–4.415], p = 0.002), and on 25 postoperative clinical variables, like arrhythmia (OR = 13.937[6.263–31.017], p<0.001), respiratory insufficiency (OR = 7.002[3.947–12.419], p<0.001), hyperkalaemia (OR = 10.378[3.909–27.555], p<0.001), nutritional supply requirement (OR = 3.576[1.894–6.752], p = 0.021), or early arthroplasty dislocation (OR = 6.557[1.206–35.640], p = 0.029). We developed a predictive model for early mortality after hip fracture surgery based on postoperative factors with 96.0% sensitivity and 60.7% specificity (AUC = 0.863).

**Conclusion:**

We revealed that not only preoperative, but also postoperative factors have a great impact after hip fracture surgery. The influence of post-operative factors on 30-day mortality has a logical basis, albeit so far they have not been identified or quantified before. Our results provide an advantageous picture of the 30-day mortality after hip fracture surgery.

## Introduction

Treatment of hip fractures in the older population is a relevant part of the healthcare activity of trauma and orthopaedic services. In the last years, many factors have raised to improve the results of hip fracture treatment, like the early surgery rates or the establishment of healthcare multidisciplinary units, the so-called orthogeriatric units [1, 2]. Despite these improvements, mortality following a hip fracture is high [3]. The early mortality rate, defined as 30-day mortality rate after hospital discharge, has been considered as an indirect indicator of the quality of care [4]. It seems to be interesting to identify the more susceptible patients to early die and thereby increase the resources and efforts for their treatment. Many studies have looked into different factors that could be associated with early mortality after hip fracture [4–8]. Previous works show also differences in the results depending on the country where the study was carried out. These differences could, in part, also reflect differences in life-expectancy and healthcare systems [9]. The most frequently early mortality related factors include biodemographic factors, like age and gender, and clinical factors, like time to surgery or comorbidities [10]. Some recently developed models assessing early mortality after hip fracture showed limited application and no postoperative approach have been assessed [11, 12]. Although these factors could be less modifiable, knowing whether they can affect the early outcome would represent a great advantage for the older hip fracture patients’ management.

The aim of this work is to analyse which factors—not only preoperative, but also postoperative and in-hospital-derived factors—could be related to 30-day mortality in patients older than 65-years-old who underwent surgery following a hip fracture at the University Hospital of Salamanca, and therefore establish a predictive model for early mortality so it could help to improve the in-hospital healthcare to this group of patients.

## Materials and methods

We design a prospective cohort study including all patients undergoing hip fracture surgery at the University Hospital of Salamanca from January 1^st^ 2018 to December 31^st^ 2019.

We included all patients over 65 with the main diagnosis of primary hip fracture. We excluded bed-ridden patients, non- surgically treated patients, and high energy trauma or tumoral aetiology fractures. In all cases, neuraxial anaesthesia was used [13]. A total of 943 patients were enrolled by signing informed consent, from whose clinical history we collected data for 130 variables. We performed on-site or telephonic 30-day follow up, noting down records for mortality. The attrition rate was 2.1%, leading a study population of 923 patients.

The study variables are grouped as biodemographic variables, like gender or place of residence; fracture-related variables, like the type of fracture or time to surgery; preoperative clinical variables, like comorbidities or admission laboratory data; and postoperative clinical variables, like surgical complications, medical complications, or walking ability at discharge.

### Ethics approval

The whole study was conducted following the Declaration of Helsinki and previously approved by the ethics committee for clinical research (CEIm) of the University Hospital of Salamanca (code: PI202001418). All participants (or their relatives) have given their written informed consent to participate.

### Statistics

Data collection was done using Microsoft^®^ Office Access 2016 (Microsoft, Inc., Redmond, WA) and data analysis was done using IBM^®^ SPSS Statics, version 25 (IBM, Inc., Armonk, NY).

Qualitative variables were analysed by contingency tables, and their statistical significance was assessed by χ^2^. Quantitative variables were analysed by mean, standard deviation, median, and range, and their statistical significance was assessed by non-parametric tests.

We performed a univariate risk assessment, estimating the odds ratio (OR) by binary logistic regression. For the multivariate analysis, we randomized patients at 50% to validate the results in half population. Seeking into previous analyses, we selected preventable candidate variables for multivariate risk assessment. We determined a predictive model for 30-days mortality, evaluated by logistic regression and ROC curve. We considered statistically significant p≤0.05 in all cases.

## Results

A total of 923 patients were enrolled and completed the 30-day follow-up. We noted a 30-day mortality rate of 6.0%, including 3.4% of in-hospital mortality rate.

Table 1 shows descriptive data for all biodemographic and fracture-related factors analysed in the study.

**Table 1:** Frequencies of biodemographic and fracture-related variables.

	Total population (n = 923)	Survival (n = 868)	30-day mortality (n = 55)	p-value
**Biodemographic variables**				
Female gender	72.9%	74.1%	54.5%	**0.002**
Age (years)	86.22 ± 6.80	86.05 ± 6.79	88.93 ± 6.48	**0.006**
	87 [65–103]	87 [65–103]	89 [73–100]	
Older than 85-years-old	64.8%	64.2%	74.5%	0.118
Older than 90-years-old	32.4%	31.3%	49.1%	**0.006**
Rural municipality (<12,500 inhabitants)	48.2%	48.6%	41.8%	0.328
Institution-living (Nursing facility)				
At admission	32.8%	33.0%	29.1%	0.550
At discharge	54.6%	54.4%	58.8%	0.612
Post-hospitalization	32.1%	31.8%	39.1%	0.458
**Fracture-related variables**				
Left side	53.0%	53.20%	54.50%	0.849
Type of fracture				0.525
Subcapital	43.2%	43.0%	47.3%	
Basicervical	4.7%	4.7%	3.6%	
Pertrochanteric	45.8%	45.7%	47.3%	
Subtrochanteric	6.3%	6.6%	1.7%	
Intracapsular fracture	53.0%	52.3%	49.1%	0.644
Surgical intervention				0.482
Osteosynthesis	55.9%	56.0%	54.5%	
Partial Hip Replacement	41.9%	41.7%	45.5%	
Total Hip Replacement	2.2%	2.3%	-	
Time to surgery (days)	2.89 ± 2.57	2.80 ± 2.46	4.33 ±3.67	**0.001**
	3 [0–23]	2 [0–23]	4 [0–20]	
Delay <24 hours	18.5%	19.2%	7.3%	**0.027**
Delay <48 hours	36.1%	36.9%	23.6%	**0.048**

Frequencies are shown in percentages and quantitative variables are described by mean ± standard deviation and median [range]. Significant differences on survival and early mortality comparison are marked in bold.

### Biodemographic variables

We noted a significantly higher rate of men in the early-mortality group (OR = 2.381 [1.371–4.136], p = 0.002), showing increasing proportion than the original population gender distribution. We also noted an increased risk for the 30-day mortality with the increasing age of patients (OR per year = 1.073 [1.025–1.122], p = 0.002), and a higher rate of patients over age 90, than patients under 90, in the 30-day mortality group (p = 0.006). We did not note significant differences regarding the place of residence or institution-living patients.

### Fracture-related variables

We did not note significant differences in the type of diagnosis or type of surgical procedure. We noted significant differences in the increasing time to surgery (OR per day = 1.183 [1.039–1146], p<0.001), noting down the protective effect for patients treated in the first 24h (OR = 0.329 [0.117–0.924], p = 0.035).

The complete analysed descriptive data for all preoperative and postoperative factors analysed in the study are on S1 Table.

### Preoperative clinical variables

Among the lab preoperative clinical variables analysed, we noted significant differences on the incidence of lymphopenia (lymphocyte count <10^3^/μl, OR = 1.842 [1.063–3.191], p = 0.029) and the albumin admission level (OR per g/dl = 0.507 [0.268–0.959], p = 0.037), establishing the critical point at albumin admission level ≤3.5g/dl (OR = 2.474 [1.316–4.643], p = 0.005).

We also noted significant differences in patients presenting chronic cardiac insufficiency (OR = 3.560 [1.955–6.482], p<0.001), previous diagnosis of arrhythmia (OR = 2.523 [1.436–4.434], p = 0.001), and previous history of ischemic heart failure (OR = 1.980 [1.012–3.873], p = 0.046). We also noted significant differences in patients taking oral anticoagulants (OR = 2.499 [1.415–4.415], p = 0.002), accented in patients treated with acenocoumarol (OR = 2.499 [1.704–5.610], p<0.001). On the other hand, we observed a decreasing incidence of daily aspirin intake (≤100mg/day) on the early mortality group, although no statistical significance was achieved (OR = 0.527 [0.254–1.093], p = 0.085).

We noted significant differences in patients with chronic obstructive pulmonary disease (OR = 2.096 [1.019–4.314], p = 0.044), and patients with a previous history of lung cancer (OR = 5.403 [1.065–27.413], p = 0.023), but no for other types of malignancy.

We also noted significant differences on ASA grade (OR = 3.273 [1.944–5.512], p<0.001) and Charlson comorbidity index (OR = 1.236[0.070–1.428], p = 0.004/High comorbidity (≥3) OR = 1.969 [1.132–3.422], p = 0.016).

### Postoperative clinical variables

#### Postoperative surgical complications

Considering the arthroplasty patients—hemiarthroplasty and total hip arthroplasty, n = 407 –, we noted significant differences on early dislocation incidence for the 30-day mortality (OR = 6.557 [1.206–35.640], p = 0.029).

#### Postoperative medical complications

We noted significant differences on many postoperative medical variables (OR = 4.050 [1.446–11.338], p = 0.008).

We noted significant differences on postoperative renal insufficiency (acute and flared-up, OR = 4.75 [2.446–9.205], p<0.001) and postoperative paralytic ileum (OR = 7.080 [1.77–28.174], p = 0.005).

We noted significant differences on cardiac complications (OR = 16.768 [9.052–31.062], p<0.001), including postsurgical arrhythmia (OR = 13.937 [6.263–31.017], p<0.001) and cardiac insufficiency (OR = 12.897 [6.525–25.488], p<0.001).

We also noted significant differences on respiratory complications (OR = 6.621 [3.769–11.631], p<0.001), including respiratory insufficiency (both acute and flared-up, OR = 7.002 [3.947–12.419], p<0.001) and upper respiratory infection (OR = 3.800 [1.499–9.635], p = 0.005).

We noted significant differences on sepsis (OR = 24.923 [4.075–152.436], p = 0.001), on postoperative nutritional supply requirement (OR = 3.576 [1.894–6.752], p = 0.021), and neurological complications (OR = 2.927 [1.629–5.258], p = 0.001), including postoperative delirium (OR = 2.565 [1.405–4.683], p = 0.002) and stroke (OR = 24.923 [4.075–152.436p<0.001).

We also noted statistical differences on metabolic ionic complications (OR = 3.287 [1.852–5.834], p<0.001), including sodium deregulation (OR = 2.929 [1.407–6.096], p = 0.004), hypernatremia (OR = 3.314 [1.092–10.056], p = 0.034) and hyponatremia (OR = 2.461 [0.997–6.076], p = 0.05), and potassium deregulation (OR = 5.238 [2.68–10.209], p<0.001), hyperkalaemia (OR = 10.378 [3.909–27.555], p<0.001) and hypokalaemia (OR = 2.934 [2.251–6.884], p = 0.013).

We also analysed in-hospital stay variables, noting a significant slight risk in the increasing length of stay (LOS) (OR per day = 1.091 [1.039–1.146], p = 0.001) and unveiling a protective factor for the walking ability at discharge (OR = 0.234 [0.127–0.432], p<0.001).

**Fig 1:**
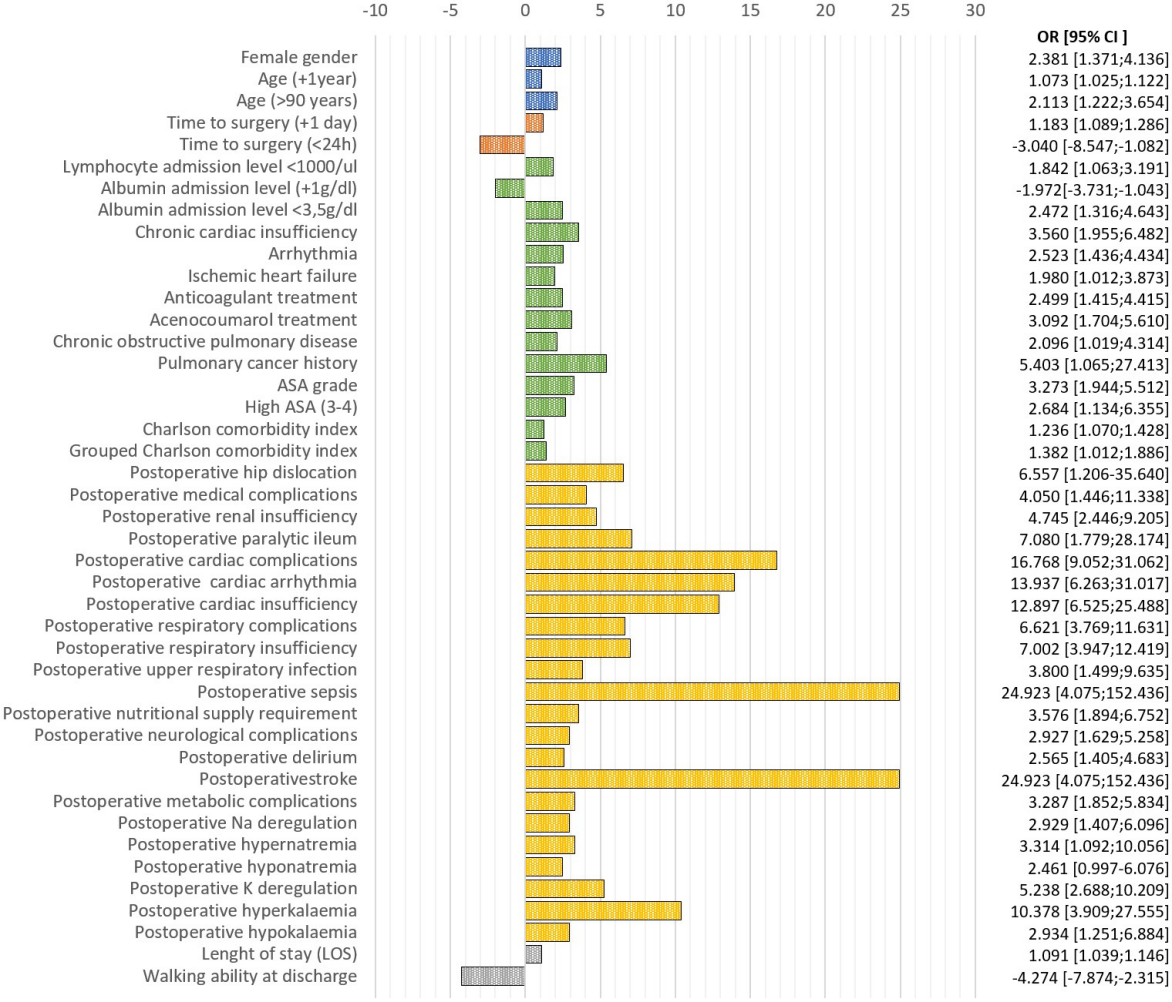
Univariate risk representation of the studied variables showing statistical significance. Blue: biodemographic variables; Red: fracture-related variables; Green: preoperative variables; Orange: postoperative variables; Grey: in-hospital stay derived variables.

Fig 1 shows an odds ratio representation on early mortality of the mentioned significant variables.

**Fig 2:**
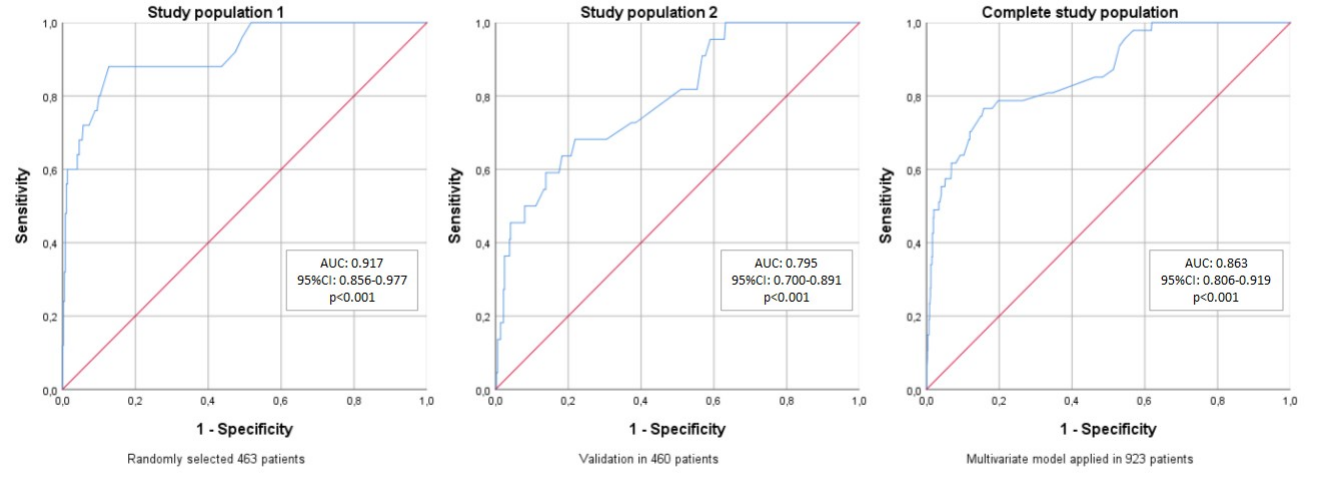
ROC curves for the developed predictive model on early mortality after hip fracture surgery, on each stage of development. AUC: Area under the curve; CI: Confidence interval.

At this stage within the study, we split the study population in half, by automatic randomization. It took 463 patients for multivariate risk assessment of selected preventable variables, and we validated the results for the 460 patients left. We could determinate a predictive model (S2 Table) that could explain 96.0% of non-early dying patients (sensitivity), and 60.7% of early dying patients (specificity), showing an AUC of 0.863 (Table 2, Fig 2).

**Table 2:** Sensibility and specificity of the multivariate model on each stage of development.

	Study population 1 n = 463	Study population 2 n = 460	Complete study population
Sensitivity	98.9%	95.2%	96.0%
Specificity	52.0%	36.4%	60.7%

The prediction equation for the developed model is as follows:

$$Prediction\;=\;\frac{1}{1+e^{-SCORE}}$$

Table 3 enables the score calculation for the prediction equation application. The mathematical expression would be as follows:

$$SCORE\;=\;-3.931{+1.479∙LAlb}_{AD}\;{+1.629∙NU}_{PO}+0.893∙{ACS}_{PO}+1.757∙{AK^{+}}_{PO}-0.906∙{RnI}_{PO}+1.429∙{RsI}_{PO}+2.568∙{CI}_{PO}\;+2.710∙{SP}_{PO}+23.228∙{ST}_{PO}{-1.597∙WA}_{PO}$$

AD: at admission; PO: Postoperative. LAlb: Lower albumin (<3.5g/dl); WA: Walking ability; NU: nutritional supply requirement; ACS: Acute confusional syndrome; AK^+^: Hyperkalaemia; RnI: Renal Insufficiency; RsI: Respiratory insufficiency; CI: Cardiac insufficiency; SP: Sepsis; ST: Stroke.

**Table 3:** Score calculation for the 30-day mortality prediction after hip fracture surgery in patients over 65.

Timeline	Variable	Record	SCORE
	Hip fracture surgical management	Yes	-3.931
Upon hospital admission	Serum albumin (g/dl)	<3.5	+1.479
		≥3.5	0
Early postoperative (before hospital discharge)	Nutritional supply requirement	Yes	+1.629
		No	0
	Acute confusional syndrome	Yes	+0.893
		No	0
	Hyperkalaemia	Yes	+1.757
		No	0
	Renal Insufficiency (acute or flared-up)	Yes	-0.906
		No	0
	Respiratory Insufficiency (acute or flared-up)	Yes	+1.429
		No	0
	Cardiac Insufficiency (acute or flared-up)	Yes	+2.568
		No	0
	Sepsis	Yes	+2.710
		No	0
	Stroke	Yes	+23.228
		No	0
	Walking ability	Yes	-1.597
		No	0
SCORE	Sum		

## Discussion

The current study analyses the influence of preoperative situation and comorbidities of older hip fracture patients on the 30-day mortality, but the main originality is that it also assesses potential early postoperative risk factors. We revealed that assessing certain postsurgical factors we could identify the frailest patients. While the postoperative risk factors assessed could be less modifiable, its identification itself represents a great advantage for the older hip fracture patients’ management. The influence of post-operative factors on early mortality has a logical basis, albeit so far they have not been identified or quantified before. This insight would support the in-hospital patient’s healthcare.

Mortality rates of hip fracture patients is a worldwide studied topic [3, 14–20]. The 30-day mortality rate after hip fracture surgery could be an indirect indicator of the in-hospital quality of care [4]. The mortality rates we report, were lower than the mean mortality rates from the Spanish National Hip Fracture Registry (SNHFR) [21], and lower than other international studies, varying from 8.25% to 13.3% for the 30-day mortality [8, 11, 22]. These variations could reflect diverse factors, some would be inherent to each patient, like inner functional reserve, and others would be inherent to the healthcare process, as the time to surgery or the complications’ management. Factors understanding could be important to concentrate efforts to avoid that excess early mortality.

### Biodemographic factors

Diverse studies have found a relationship between hip fracture mortality rate with age and gender [8, 23, 24], showing that the 30-day mortality rate after hip fracture is higher in men and oldest patients. We corroborate it, noting down the over 85 mean age of our population.

### Fracture-related factors

We found significantly increased time to surgery in patients who early died, what is consistent with many other studies backing up the idea that early surgery could avoid the early mortality [4, 22, 25–27]. However, it is difficult to consider the early surgery as a protective factor by itself, as this could not be achieved the more complex patients with poor previous medical conditions that may be playing as confounder factors. We consider maybe the risk factor previously assessed would be rather the late surgery than the early surgery. As our mean time to surgery never excess the five days, fewer patients underwent late surgery.

### Preoperative clinical factors

We noticed that the lower lymphocyte count and albumin admission levels were associated with the 30-day mortality. It could be also related to those patients who will require nutritional supply and reveal the importance of the good nutritional status of older patients [28, 29].

We also noted a negative effect of the anticoagulant treatment. Patients suffering from chronic cardiac insufficiency, previous diagnosis of arrhythmia, or previous history of ischemic heart failure use to take anticoagulant treatment. The anticoagulant treatment always carries a higher time to surgery and could be a reason why we noted an increased time to surgery on early dying patients. Rutenberg et al. [30] also showed a relationship between anticoagulant treatment and increased time to surgery for hip fracture, but they did not associate the anticoagulant treatment with the one-year mortality. Nonetheless, other previous studies also showed an increased time to surgery but also decreased survival [31–33].

Further, we recorded the Charlson comorbidity index, which showed a positive correlation with the 30-day mortality, as well as ASA grade. Both clinical scores assess high 30-day mortality in the worst clinical stated patients, what is consistent, and reveals the importance of the clinical status previous to hip fracture [24, 34].

### Postoperative clinical factors

Regarding the surgical-derived complications, we found the hip dislocation as a relevant risk factor for early mortality. Some authors already noted that most hip dislocations occurred within the first month after surgery [35–37]. Nonetheless, the incidence of this complication is varying from <1% to 3.5% [35, 37, 38]. Recently, Kishimoto et al. [39] pointed dislocation as a risk factor for long term survival after total hip arthroplasty, but only one case was a primary hip fracture. On the other hand, there are other works defeating no increase of mortality following a hip dislocation [35, 36]. Also, the prosthesis dislocation management may lead to bias for the results presented. Of the 407 patients who received hip arthroplasty in our study, only seven patients suffered a hip dislocation. Taking account of the low incidence of this complication in our study, we would consider a greater population to validate this finding.

On the medical postoperative complications, sepsis was the analysed risk factor showing the highest OR. Although sepsis incidence was slight, it is concordant that patient’s sepsis would carry a worse outcome. A similar upshot was revealed for the stroke [24]. The postoperative delirium is also a risk factor validated in this study, as previously pointed Mosk et al. [40] for later mortality.

We noted a higher incidence of sodium and potassium postoperative misbalance on the early mortality group, unexpected relevant risk factors not assessed before. It all raises the importance of metabolic monitoring for older patients undergoing hip fracture surgery. They could be also related to renal insufficiency, which also was assessed as a risk factor for early mortality, so those complications should be taken in high consideration, even with cardiac [41] and respiratory complications.

The ability to walk at discharge is a functional variable that seems to be a protective factor for the early mortality after hip fracture surgery, which agrees with its recent definition as a predictive factor for long-term survival [42].

Most studies already cited focused on pre-operative risk factors to assess the early mortality after hip fracture surgery, but we should consider the whole in-hospital process, including the immediate postoperative management, so it will significantly affect the patient’s outcome. We developed a novel early mortality prediction model, in which we only included preventable variables. Preventable variables were defined as those variables that are not inherent to the patient, but physicians could modify and take into consideration in order to prevent or motivate. Our model has a 96.0% sensitivity and 60.7% specificity, and AUC of 0.863, which validates it for further consideration on larger populations. It would allow us to early detect the frailest patients who underwent hip fracture surgery so we could assess an earlier follow-up to prevent early mortality.

There are other recently developed models assessing early mortality after hip fracture. Karres et al. [11] evaluated six different predictive models, some of them extensively used (as NHFS or O-Possum), and none of them showed excellent discrimination, as their AUC always were less than 0.8. The most recently published early mortality predictive model for hip fracture is the Brabant Hip Fracture Score [12], but no postoperative approach was assessed.

In conclusion, we found significant preoperative risk factors for early mortality after hip fracture, but the postoperative risk factors revealed a higher impact on the patient’s outcome at 30-days. In this sense, it is necessary to focus our efforts to decrease the postoperative complications rate on those patients in order to avoid the early mortality.

While the postoperative risk factors assessed could be less modifiable, its identification itself represents a great advantage for the older hip fracture patients’ management. This insight would support the in-hospital patient’s healthcare.

Besides our discussion, the study has some limitations as we studied single-center operated patients. Our upshot would be better considering a greater population.

## Supporting information

S1 Table Complete analysed descriptive data for all preoperative and postoperative factors analysed in the study population, and its comparison between the 30-day mortality group and survival group. (PDF)

S2 Table Definition of parameters in the equation for the predictive model assessing the 30-day mortality of hip fracture patients. (PDF)

## References

[1] Kristensen PK, Thillemann TM, Søballe K, Johnsen SP. Can improved quality of care explain the success of orthogeriatric units? A population-based cohort study. Age Ageing. 2016;45: 66–71. 10.1093/ageing/afv155 26582757

[2] Giannoudis V, Panteli M, Giannoudis P V. Management of polytrauma patients in the UK: Is there a ‘weekend effect’? Injury. 2016;47: 2385–2390. 10.1016/j.injury.2016.10.007 27809989

[3] Katsoulis M, Benetou V, Karapetyan T, Feskanich D, Grodstein F, Pettersson-Kymmer U, et al Excess mortality after hip fracture in elderly persons from Europe and the USA: the CHANCES project. J Intern Med. 2017;281: 300–310. 10.1111/joim.12586 28093824

[4] Khan MA, Hossain FS, Ahmed I, Muthukumar N, Mohsen A. Predictors of early mortality after hip fracture surgery. Int Orthop. 2013;37: 2119–2124. 10.1007/s00264-013-2068-1 23982637 PMC3824905

[5] Forni C, Gazineo D, D’Alessandro F, Fiorani A, Morri M, Sabattini T, et al Predictive factors for thirty day mortality in geriatric patients with hip fractures: a prospective study. Int Orthop. 2019;43: 275–281. 10.1007/s00264-018-4057-x 30054670

[6] Sheikh HQ, Hossain FS, Aqil A, Akinbamijo B, Mushtaq V, Kapoor H. A Comprehensive Analysis of the Causes and Predictors of 30-Day Mortality Following Hip Fracture Surgery. Clin Orthop Surg. 2017;9: 10–18. 10.4055/cios.2017.9.1.10 28261422 PMC5334018

[7] Nijmeijer WS, Folbert EC, Vermeer M, Slaets JP, Hegeman JH. Prediction of early mortality following hip fracture surgery in frail elderly: The Almelo Hip Fracture Score (AHFS). Injury. 2016;47: 2138–2143. 10.1016/j.injury.2016.07.022 27469403

[8] Hu F, Jiang C, Shen J, Tang P, Wang Y. Preoperative predictors for mortality following hip fracture surgery: A systematic review and meta-analysis. Inj Int J Care Inj. 2012;43: 676–685. 10.1016/j.injury.2011.05.017 21683355

[9] Nikkel LE, Kates SL, Schreck M, Maceroli M, Mahmood B, Elfar JC. Length of hospital stay after hip fracture and risk of early mortality after discharge in New York state: retrospective cohort study. BMJ. 2015;351: h6246 10.1136/bmj.h6246 26655876 PMC4674667

[10] Yong E-L, Ganesan G, Kramer MS, Howe Sen T, Koh JSB, Thu WP, et al Risk Factors and Trends Associated With Mortality Among Adults With Hip Fracture in Singapore. JAMA Netw Open. 2020;3: e1919706 10.1001/jamanetworkopen.2019.19706 32058551 PMC12124694

[11] Karres J, Heesakkers NA, Ultee JM, Vrouenraets BC. Predicting 30-day mortality following hip fracture surgery: Evaluation of six risk prediction models. Injury. 2015;46: 371–377. 10.1016/j.injury.2014.11.004 25464983

[12] van de Ree CL, Gosens T, van der Veen AH, Oosterbos CJ, Heymans MW, de Jongh MA. Development and validation of the Brabant Hip Fracture Score for 30-day and 1-year mortality. Hip Int. 2020;30: 354–362. 10.1177/1120700019836962 30912455

[13] Maxwell BG, Spitz W, Porter J. Association of Increasing Use of Spinal Anesthesia in Hip Fracture Repair With Treating an Aging Patient Population. JAMA Surg. 2020;155: 167 10.1001/jamasurg.2019.4471 31746959 PMC6902104

[14] Empana J-P, Dargent-Molina P, Breart G, EPIDOS Group. Effect of Hip Fracture on Mortality in Elderly Women: The EPIDOS Prospective Study. J Am Geriatr Soc. 2004;52: 685–690. 10.1111/j.1532-5415.2004.52203.x 15086646

[15] Kanis JA, Oden A, Johnell O, De Laet C, Jonsson B, Oglesby AK. The components of excess mortality after hip fracture. Bone. 2003;32: 468–473. 10.1016/s8756-3282(03)00061-9 12753862

[16] Haentjens P, Magaziner J, Colón-Emeric CS, Vanderschueren D, Milisen K, Velkeniers B, et al Meta-analysis: Excess Mortality After Hip Fracture Among Older Women and Men. Ann Intern Med. 2010;152: 380 10.7326/0003-4819-152-6-201003160-00008 20231569 PMC3010729

[17] Grønskag AB, Romundstad P, Forsmo S, Langhammer A, Schei B. Excess mortality after hip fracture among elderly women in Norway. Osteoporos Int. 2012;23: 1807–1811. 10.1007/s00198-011-1811-y 22068386

[18] Farahmand BY, Michaëlsson K, Ahlbom A, Ljunghall S, Baron JA, Swedish Hip Fracture Study Group. Survival after hip fracture. Osteoporos Int. 2005;16: 1583–1590. 10.1007/s00198-005-2024-z 16217590

[19] Richmond J, Aharonoff GB, Zuckerman JD, Koval KJ. Mortality risk after hip fracture. J Orthop Trauma. 2003;17: 53–6. 10.1097/00005131-200301000-00008 12499968

[20] Menéndez-Colino R, Alarcon T, Gotor P, Queipo R, Ramírez-Martín R, Otero A, et al Baseline and pre-operative 1-year mortality risk factors in a cohort of 509 hip fracture patients consecutively admitted to a co-managed orthogeriatric unit (FONDA Cohort). Injury. 2018;49: 656–661. 10.1016/j.injury.2018.01.003 29329713

[21] Ojeda-Thies C, Sáez-López P, Currie CT, Tarazona-Santalbina FJ, Alarcón T, Muñoz-Pascual A, et al Spanish National Hip Fracture Registry (RNFC): analysis of its first annual report and international comparison with other established registries. Osteoporos Int. 2019;30: 1243–1254. 10.1007/s00198-019-04939-2 30904929

[22] Pincus D, Ravi B, Wasserstein D, Huang A, Paterson JM, Nathens AB, et al Association Between Wait Time and 30-Day Mortality in Adults Undergoing Hip Fracture Surgery. JAMA. 2017;318: 1994 10.1001/jama.2017.17606 29183076 PMC5820694

[23] Liu Y, Wang Z, Xiao W. Risk factors for mortality in elderly patients with hip fractures: a meta-analysis of 18 studies. Aging Clin Exp Res. 2018;30: 323–330. 10.1007/s40520-017-0789-5 28660596

[24] Durand WM, Goodman AD, Johnson JP, Daniels AH. Assessment of 30-day mortality and complication rates associated with extended deep vein thrombosis prophylaxis following hip fracture surgery. Injury. 2018;49: 1141–1148. 10.1016/j.injury.2018.03.019 29580646

[25] Beaupre LA, Khong H, Smith C, Kang S, Evens L, Jaiswal PK, et al The impact of time to surgery after hip fracture on mortality at 30- and 90-days: Does a single benchmark apply to all? Injury. 2019;50: 950–955. 10.1016/j.injury.2019.03.031 30948037

[26] Khan SK, Kalra S, Khanna A, Thiruvengada MM, Parker MJ. Timing of surgery for hip fractures: A systematic review of 52 published studies involving 291,413 patients. Injury. 2009;40: 692–697. 10.1016/j.injury.2009.01.010 19450802

[27] Lewis PM, Waddell JP. When is the ideal time to operate on a patient with a fracture of the hip? Bone Joint J. 2016;98-B: 1573–1581. 10.1302/0301-620X.98B12.BJJ-2016-0362.R2 27909117

[28] Bohl DD, Shen MR, Hannon CP, Fillingham YA, Darrith B, Della Valle CJ. Serum Albumin Predicts Survival and Postoperative Course Following Surgery for Geriatric Hip Fracture. J Bone Joint Surg Am. 2017;99: 2110–2118. 10.2106/JBJS.16.01620 29257017

[29] Wilson JM, Lunati MP, Grabel ZJ, Staley CA, Schwartz AM, Schenker ML. Hypoalbuminemia Is an Independent Risk Factor for 30-Day Mortality, Postoperative Complications, Readmission, and Reoperation in the Operative Lower Extremity Orthopaedic Trauma Patient. J Orthop Trauma. 2019;33: 284–291. 10.1097/BOT.0000000000001448 30720559

[30] Frenkel Rutenberg T, Velkes S, Vitenberg M, Leader A, Halavy Y, Raanani P, et al Morbidity and mortality after fragility hip fracture surgery in patients receiving vitamin K antagonists and direct oral anticoagulants. Thromb Res. 2018;166: 106–112. 10.1016/j.thromres.2018.04.022 29727737

[31] Novoa-Parra CD, Hurtado-Cerezo J, Morales-Rodríguez J, Sanjuan-Cerveró R, Rodrigo-Pérez JL, Lizaur-Utrilla A. Factores predictivos de la mortalidad al año en pacientes mayores de 80 años intervenidos de fractura del cuello femoral. Rev Esp Cir Ortop Traumatol. 2019;63: 202–208. 30795998 10.1016/j.recot.2018.10.007

[32] Lawrence JE, Fountain DM, Cundall-Curry DJ, Carrothers AD. Do Patients Taking Warfarin Experience Delays to Theatre, Longer Hospital Stay, and Poorer Survival After Hip Fracture? Clin Orthop Relat Res. 2017;475: 273–279. 10.1007/s11999-016-5056-0 27586655 PMC5174047

[33] López S, da Casa C, Pablos-Hernández C, Pescador D, Díaz-Álvarez A, Asensio N, et al The impact of antithrombotic therapy on surgical delay and 2-year mortality in older patients with hip fracture: a prospective observational study. Eur Geriatr Med. 2020;11: 555–561. 10.1007/s41999-020-00293-3 32297256

[34] Smith T, Pelpola K, Ball M, Ong A, Myint PK. Pre-operative indicators for mortality following hip fracture surgery: a systematic review and meta-analysis. Age Ageing. 2014;43: 464–471. 10.1093/ageing/afu065 24895018

[35] Gill JR, Kiliyanpilakkill B, Parker MJ. Management and outcome of the dislocated hip hemiarthroplasty. Bone Joint J. 2018;100-B: 1618–1625. 10.1302/0301-620X.100B12.BJJ-2018-0281.R1 30499311

[36] Salem K, Shannak O, Scammell B, Moran C. Predictors and outcomes of treatment in hip hemiarthroplasty dislocation. Ann R Coll Surg Engl. 2014;96: 446–451. 10.1308/003588414X13946184903045 25198977 PMC4474197

[37] Jones C, Briffa N, Jacob J, Hargrove R. The Dislocated Hip Hemiarthroplasty: Current Concepts of Etiological factors and Management. Open Orthop J. 2017;11: 1200–1212. 10.2174/1874325001711011200 29290857 PMC5721319

[38] Hansson S, Bülow E, Garland A, Kärrholm J, Rogmark C. More hip complications after total hip arthroplasty than after hemi-arthroplasty as hip fracture treatment: analysis of 5,815 matched pairs in the Swedish Hip Arthroplasty Register. Acta Orthop. 2020;91: 133–138. 10.1080/17453674.2019.1690339 31735103 PMC7144190

[39] Kishimoto Y, Kato Y, Kishi T, Takahashi T, Kuranobu K. Dislocation is a Leading Cause of Decreased Survival Rate in Primary Total Hip Arthroplasty Performed by Low-Volume Surgeons: Long-Term Retrospective Cohort Study. J Arthroplasty. 2020;Article in: S0883-5403(20)30605–7. 10.1016/j.arth.2020.05.064 32586657

[40] Mosk C, Mus M, Vroemen J, van der Ploeg T, Vos D, Elmans L, et al Dementia and delirium, the outcomes in elderly hip fracture patients. Clin Interv Aging. 2017;12: 421–430. 10.2147/CIA.S115945 28331300 PMC5354532

[41] Lee R, Lee D, Gowda NB, Probasco W V, Ibrahim G, Falk DP, et al Surgical complications associated with congestive heart failure in elderly patients following primary hip hemiarthroplasty for femoral neck fractures. Eur J Orthop Surg Traumatol. 2019;Epub ahead. 10.1007/s00590-019-02438-y 31041543

[42] Iosifidis M, Iliopoulos E, Panagiotou A, Apostolidis K, Traios S, Giantsis G. Walking ability before and after a hip fracture in elderly predict greater long-term survivorship. J Orthop Sci. 2016;21: 48–52. 10.1016/j.jos.2015.09.009 26755386

