# Supplementary material for: 30-day mortality after hip fracture surgery: Influence of postoperative factors
Source: PLoS One. 2021 Feb 16;16(2):e0246963. doi: 10.1371/journal.pone.0246963 (PMC7886122; doi:10.1371/journal.pone.0246963)
Supplement: S1 Table — (PDF) [file pone.0246963.s001.pdf]

30-day mortality after hip fracture surgery: influence of postoperative factors.

Juan F Blanco, Carmen da Casa, Carmen Pablos-Hernández, Alfonso González-Ramírez, José Miguel Julián-Enríquez, Agustín Díaz-Álvarez

S1 Table. Descriptive data for pre-operative and post-operative studied variables including statistical comparisons between survival and 30-day mortality groups.

	**Total population** (n=923)	**Survival** (n=868)	**30-day mortality** (n=55)	**p-value**
	**In-hospital stay derived variables**			
**LOS (days)**	8.65 ± 4.09<br>8 [1,37]	8.53 ± 3.82<br>8 [2,37]	10.58 ± 6.87<br>8 [1,34]	0.099
**Walking ability at discharge**	60.1%	62.1%	27.8%	**<0.001**
	**Pre-surgical clinical variables**			
**ASA**	2.84 ± 0.56<br>3 [1,4]	2.81 ± 0.56<br>3 [1,4]	3.16 ± 0.60<br>3 [2,4]	**<0.001**
**ASA 3-4**	76.1%	75.3%	89.1%	**0.020**
**Charlson index**	1.86 ± 1.64<br>2 [0,10]	1.82 ± 1.61<br>2 [0,10]	2.49 ± 1.98<br>2 [0,8]	**0.016**
**Charlson index (grouped)**				**0.049**
No comorbidity (0-1)	48.6%	49.2%	40.0%	
Slight comorbidity (2)	22.2%	22.6%	16.4%	
High comorbidity (≥3)	29.1%	28.2%	43.9%	**0.015**
**Cognitive impairment**	41.2%	40.4%	52.8%	0.075
**Antiplatelet treatment**	34.5%	34.9%	27.3%	0.248
Acetyl salicylic acid (≤100mg/day)	26.4%	27.1%	16.4%	0.081
Acetyl salicylic acid (300mg/day)	3.1%	3.0%	5.5%	0.311
Clopidogrel	4.0%	4.0%	5.4%	0.885
Other strong antiplatelet agent	1.8%	1.8%	1.8%	0.989
**Anticoagulant treatment**	20.9%	19.8%	38.2%	**0.001**
Acenocoumarol	14.7%	13.6%	32.7%	**<0.001**
Other DOACs	6.3%	6.3%	5.5%	0.794
**Comorbidity**	93.7%	93.5%	96.4%	0.404
**Cardiac comorbidity**	87.5%	87.3%	90.9%	0.435
High blood pressure	74.8%	75.0%	70.9%	0.498
Dyslipidaemia	39.2%	39.3%	36.4%	0.662
Chronic cardiac insufficiency	13.3%	12.0%	14.8%	**<0.001**
Valvar heart disease	9.4%	9.0%	16.4%	0.070
Arrhythmia	22.0%	20.9%	40.0%	**0.001**
Ischemic heart failure	12.9%	12.4%	21.8%	**0.042**

	Total population (n=923)	Survival (n=868)	30-day mortality (n=55)	p-value
Diabetes	26.8%	26.8%	27.3%	0.938
**Pulmonary comorbidity**	17.9%	17.7%	21.8%	0.436
COPD	10.1%	9.6%	18.2%	**0.040**
Pulmonary emphysema	0.8%	0.7%	1.8%	0.351
Pulmonary thromboembolism	3.5%	3.5%	3.6%	0.946
Asthma	5.4%	5.4%	5.5%	0.993
**Digestive comorbidity**	28.5%	28.6%	27.3%	0.832
Hiatal hernia	6.0%	5.7%	10.9%	0.111
Chronic gastritis	1.8%	2.0%	-	0.294
Diverticulitis	5.0%	4.8%	7.3%	0.424
Gastric ulcus or Duodenal ulcus	4.3%	4.4%	3.6%	0.791
Gastroesophageal reflux	1.4%	1.4%	1.8%	0.792
Chronic diarrhoea	0.2%	0.2%	-	0.721
Other hepatic-pancreatic-biliary comorbidity	15.8%	16.3%	7.3%	0.075
**Renal comorbidity**	16.7%	16.4%	21.8%	0.296
Chronic renal insufficiency	14.7%	14.2%	21.8%	0.122
Recurrent urinary tract infections	2.7%	2.9%	-	0.201
**History of malignancy**	23.0%	22.6%	29.1%	0.270
Digestive cancer	5.2%	5.1%	7.3%	0.478
Renal cancer	5.0%	5.0%	5.5%	0.872
Pulmonary cancer	0.9%	0.7%	3.6%	**0.023**
Gynaecological cancer	3.9%	4.0%	2.8%	0.409
Endocrine cancer	0.2%	0.1%	1.8%	**0.009**
Haematological cancer	1.3%	1.3%	1.8%	0.729
Other previous cancer	9.1%	9.1%	9.1%	0.994
**Post-surgical clinical variables**				
**Surgical-derived complications**	25.9%	26.2%	21.8%	0.473
**In-hospital surgical-derived complications**	23.2%	23.4%	20.0%	0.562
**Seroma**	20.5%	20.7%	16.4%	0.435
**Intraoperative fracture**	0.4%	0.5%	-	0.613
**Out-of-hospital surgical-derived complications**	3.6%	3.6%	3.6%	0.986
**Reoperation**				0.097
Cut in	0.2%	0.2%	-	
Cut out	2.0%	2.1%	-	
Dislocation*	1.7%	1.3%	8.0%	**0.013**

	Total population (n=923)	Survival (n=868)	30-day mortality (n=55)	p-value
**Prosthesis infection**	0.9%	0.9%	-	0.473
**Medical complication**	76.9%	75.9%	92.7%	**0.004**
**Renal complication**	33.8%	33.1%	45.5%	0.060
Postsurgical renal insufficiency (grouped)	7.8%	6.7%	25.5%	**<0.001**
Acute renal insufficiency	4.6%	4.1%	12.7%	**<0.001**
Chronic renal insufficiency	3.3%	2.7%	12.7%	
Acute urine retention	27.5%	27.8%	23.6%	0.505
Urine tract infection	3.4%	3.5%	1.8%	0.509
**Digestive complications**	24.8%	24.55	29.1%	0.448
Constipation	23.5%	23.4%	25.5%	0.725
Diarrhoea	0.5%	0.6%	-	0.571
Paralytic ileum	1.1%	0.8%	5.5%	**0.001**
Upper digestive haemorrhage	0.4%	0.3%	1.8%	0.108
Lower digestive haemorrhage	0.4%	0.3%	-	0.662
Hepatic-pancreatic-biliary complication	0.4%	0.3%	1.8%	0.108
**Cardiac complication**	7.2%	4.7%	45.5%	**<0.001**
Arrhythmia	3.1%	2.0%	21.8%	**<0.001**
Cardiac insufficiency	5.0%	3.4%	30.9%	**<0.001**
Acute heart failure	0.3%	0.3%	-	0.662
**Respiratory complication**	15.8%	13.5%	19.3%	**<0.001**
Postsurgical respiratory insufficiency (grouped)	12.7%	10.6%	45.5%	**<0.001**
Acute respiratory insufficiency	10.2%	8.2%	41.8%	**<0.001**
Chronic respiratory insufficiency	2.5%	2.4%	3.7%	
Upper respiratory infection	3.6%	3.1%	10.9%	**0.003**
Pneumonia	0.7%	0.6%	1.8%	0.267
Pulmonary thromboembolism	0.2%	0.1%	1.8%	**0.009**
**Postsurgical sepsis**	0.5%	0.2%	5.5%	**<0.001**
**Nutritional requirement**	10.6%	9.5%	27.3%	**<0.001**
**Postsurgical dysphagia**	1.6%	1.5%	3.6%	0.227
**Diabetic decompensation**	4.2%	4.0%	7.3%	0.249
**Postsurgical pressure ulcer**	1.4%	1.4%	1.8%	0.794
**Postsurgical venous thrombosis**	-	-	-	

	Total population (n=923)	Survival (n=868)	30-day mortality (n=55)	p-value
**Postsurgical blood transfusion**	39.0%	39.4%	32.7%	0.324
**Postsurgical intravenous iron administration**	7.8%	7.6%	10.9%	0.383
**Neurological complication**	16.4%	15.3%	34.5%	**<0.001**
Acute confusional syndrome	15.8%	14.8%	30.9%	**0.002**
Stroke	0.5%	0.2%	5.5%	**<0.001**
Convulsions	6.0%	6.0%	-	0.662
Subdural hematoma	-	-	-	
**Metabolic misbalance**	17.2%	15.8%	38.2%	**<0.001**
**Sodium deregulation**	7.7%	7.1%	18.2%	**0.003**
Hypernatremia	2.6%	2.3%	7.3%	**0.025**
Hyponatremia	5.1%	4.7%	10.9%	**0.044**
**Potassium deregulation**	7.3%	6.1%	25.5%	**<0.001**
Hyperkalaemia	5.2%	4.7%	12.7%	**<0.001**
Hypokalaemia	6.8%	6.6%	10.9%	**0.010**
**Calcium complication** (hypercalcaemia)	6.8%	6.6%	10.9%	0.218
Hypocalcaemia	-	-	-	
		**Laboratory variables**		
**Admission haemoglobin (g/dl)**	12.62 ± 1.72<br>12.7 [5.6,17.5]	12.62 ± 1.68<br>12.7 [5.6,17.5]	12.585 ± 2.28<br>12.5 [8.0,17.5]	0.368
Haemoglobin level ≤10g/dl	6.6%	6.5%	8.2%	0.448
**Admission lymphocyte count ($x10^3/\mu l$)**	1.28 ± 0.92<br>1.1 [0.25,16.15]	1.28 ± 0.84<br>1.13 [0.25,16.15]	1.31 ± 1.74<br>0.93 [0.32,13.09]	**0.019**
**Admission lymphocyte count (grouped)**				0.079
Lymphocyte level <0.5 $x10^3/\mu l$	5.5%	5.3%	9.1%	
Lymphocyte level <1 $x10^3/\mu l$	42.1%	41.2%	56.4%	**0.027**[a]
Lymphocyte level 0.5-1 $x10^3/\mu l$	36.6%	35.9%	47.3%	
Lymphocyte level 1-2.5 $x10^3/\mu l$	52.9%	53.9%	36.4%	
Lymphocyte level >2.5 $x10^3/\mu l$	5.0%	4.8%	8.7%	
**Admission Albumin (g/dl)**	3.92 ± 0.43<br>4 [2.3,5.1]	3.93 ± 0.42<br>4 [2.3,5.1]	3.80 ± 0.50<br>3.85 [2.8,4.8]	0.061
**Admission Albumin (grouped)**				**0.026**
Albumin level <3.5 g/dl	17.8%	16.8%	33.3%	**0.009**[b]
Albumin level 3.5-4 g/dl	41.1%	41.7%	33.3%	

	Total population (n=923)	Survival (n=868)	30-day mortality (n=55)	p-value
Albumin level 4-4.5 g/dl	36.6%	37.2%	27.1%	
Albumin level >4.5 g/dl	4.5%	4.3%	6.3%	**0.049**
**Admission glucose (mg/dl)**	143.5 ± 49.0<br>132 [65,628]	149.0 ± 49.5<br>131 [65,628]	146.0 ± 41.2<br>136 [84,263]	0.414
**Admission glucose (grouped)**				0.928
Glucose level <100 mg/dl	8.0%	8.0%	7.5%	
Glucose level >110mg/dl	78.9%	78.7%	83.0%	0.450[c]
Glucose level 100-120 mg/dl	26.3%	26.6%	22.6%	
Glucose level 120-140 mg/dl	27.1%	27.0%	28.3%	
Glucose level >140 mg/dl	38.6%	38.4%	41.5%	
**Parathyroid hormone (mg/dl)**[d]	72.95 ± 52.05<br>59.6 [6.4,437.6]	71.97 ± 48.09<br>59.6 [6.4,371]	90.08 ± 99.3<br>72.6 [13.9,437.6]	0.919
**Vitamin D (mg/dl)**[e]	12.11 ± 8.83<br>9.15 [1.0,57.7]	12.06 ± 8.69<br>9.1 [1.0,57.7]	13.06 ± 11.50<br>10.1 [3.0,40.2]	0.947
**Discharge lymphocyte count ($x10^3/\mu l$)**	1.24 ± 0.76<br>1.10 [0.16,14.06]	1.24 ± 0.69<br>1.11 [0.16,14.06]	1.26 ± 1.54<br>0.93 [0.22-10.1]	**0.006**
**Discharge albumin (g/dl)**[f]	3.02 ± 0.40<br>3.0 [1.6,4.7]	3.01 ± 0.38<br>3.0 [1.6,4.7]	3.17 ± 0.61<br>3.05 [1.9,4.5]	0.262

LOS: Length of stay; DOACs: Direct Oral Anticoagulants. Frequencies are shown in percentages and quantitative variables are described by mean and standard deviation. P values are indicating differences between survival and 30-day mortality groups. Significant differences are marked in bold. *Analysis include only arthroplasty patients (n=407, n=382 survival, n=25 30-day mortality). a: Comparison of lymphocyte count $<10^3/\mu l$ vs. $\geq 10^3/\mu l$. b: comparison of albumin admission level <3.5g/dl vs. ≥3.5g/dl. c: comparison of glucose admission level >110mg/dl vs. ≤110mg/dl. d: missing data, n=313 (n=17 30-day mortality). e: missing data, n=302 (n=15 30-day mortality). f: missing data, n=439.
