# Supplementary material for: 30-day mortality after hip fracture surgery: Influence of postoperative factors
Source: PLoS One. 2021 Feb 16;16(2):e0246963. doi: 10.1371/journal.pone.0246963 (PMC7886122; doi:10.1371/journal.pone.0246963)
Supplement: S2 Table — (PDF) [file pone.0246963.s002.pdf]

30-day mortality after hip fracture surgery: influence of postoperative factors.

Juan F Blanco, Carmen da Casa, Carmen Pablos-Hernández, Alfonso González-Ramírez, José Miguel Julián-Enríquez, Agustín Díaz-Álvarez

S2 Table. Variables on the equation for the predictive model on 30-day mortality.

	B	Exp(B)	EXP(B) 95% CI		Wald	p-value
			Lower	Upper		
**Albumin admission level <3.5g/dl**	1.479	4.389	1.301	14.808	5.684	0.017
**Walking ability at discharge**	-1.597	0.203	0.057	0.724	6.036	0.014
**Nutritional supply requirement**	1.629	5.100	1.522	17.092	6.972	0.008
**Post-surgical acute confusional syndrome**	0.893	2.441	0.700	8.520	1.959	0.162
**Post-surgical hyperkalaemia**	1.757	5.795	0.525	63.975	2.056	0.152
**Post-surgical renal insufficiency**	-0.906	0.404	0.066	2.464	.965	0.326
**Post-surgical respiratory insufficiency**	1.429	4.175	1.273	13.686	5.565	0.018
**Post-surgical cardiac insufficiency**	2.568	13.040	3.516	48.358	14.747	0.000
**Post-surgical sepsis**	2.710	15.022	0.786	287.261	3.239	0.072
**Post-surgical stroke**	23.228	1.224e10	0.000	.	.000	0.999
**Constant**	-3.931				57.155	0.000

B: coefficient in equation. CI: confidence interval.
